# Buffalo Medical Association

**Published:** 1879-11

**Authors:** 


### Society Reports.

### BUFFALO MEDICAL ASSOCIATION.

*Stated Meeting, October 7, 1879.*

DR. LUCIEN HOWE, PRESIDENT, IN THE CHAIR.

Members present, Drs. Howe, Trowbridge, Samo, Moody, Hauenstein, White, Rochester, Cary, Johnson, Hartwig, Habenstreit, Bartlett, Keene, Lothrop, Fowler, and, on invitation, Dr. Granger.

Dr. Mary B. Moody read an interesting paper on Placenta Prævia.

DISCUSSION.

Prof. White said he felt under obligations to his professional sister, for presenting the subject of placenta prævia so intelligently before the Association. Dr. White's remarks were confined to the treatment.

This unnatural birth is no longer left to Nature alone, but placed in the hands of art and successfully managed in the great majority of cases.

The plan recommended in the paper was that which he had practiced himself for a number of years, and which he believed was a great step forward in the treatment of placenta prævia and truly an American achievement.

In his instruction to his classes, he always urged that these cases be kept under the eye of the practitioner, when the presence of hemorrhage during gestation had admonished him of his danger. As soon as the child shows signs of viability, and the safety of the mother warrants, labor should be brought on. This had been called meddlesome midwifery. It is the surest way of protecting both the mother and child.

The life of the child would not necessarily be sacrificed at seven and one-half or eight months. It would be more liable to live, if delivered at this period, than at full time when profuse hemorrhage had preceded its birth, and the child delivered exsanguine.

Dr. White said he did not remember whether Dr. Thomas or himself practiced this method first. He remembered a case in which Dr. Rochester did him the honor to invite his counsel and assistance. An occasional hemorrhage during the last months of pregnancy led Dr. R. to suspect the presence of placenta prævia.

As soon as it was possible, they proceeded to deliver, which was accomplished without the loss of much blood. The patient sank back exhausted, and died a few minutes later. It was then ascertained that just previous to their visit, she entered the closet to urinate, and, while seated, passed what was believed to be liquor amnii, but what subsequent investigation proved to be a large amount of blood. If premature labor had been brought on early, her life would have been saved.

Another case he examined in consultation with Dr. Hauenstein. This case was reported in the last number of the JOURNAL.

Dr. Rochester remarked that he had met with eight cases in thirty-one years' practice. He spoke of a case of placenta prævia lateralis occurring about a year ago. When the lady had advanced seven and one-half months in pregnancy, he determined to induce labor. He was summoned hastily to see her, and found she had already lost considerable blood, and the hemorrhage still going on. He at once prepared to turn and deliver. He found the placenta partially detached. With little manipulation the head presented, the membranes ruptured, and the child was born dead. The lady made a good recovery.

Another case occurred about eight months ago. The patient's husband came in great haste, saying his wife was flowing badly, and he was afraid she would be dead on his return. He found her pale, almost pulseless, having lost an enormous amount of blood. Applied the forceps at once, gave ergot, and delivered her of a large lifeless child. Her husband was directed to press firmly on the uterus with both hands, which he did, after the child was born. The placenta fell out of its own accord, and the lady recovered.

Dr. Hauenstein said he presumed it would be expected that he should make some remarks upon this subject, having gained a considerable local notoriety in consequence of the frequency of his cases, numbering in all eleven, and what seems most remarkable and almost incredible to the profession, six of these occurred in a period of eight months. What he should say would be confined to the manner of treatment. So many methods having been proposed and recommended, physicians are confused if suddenly called upon to treat a case. He thought it best to be familiar with some definite plan of treatment. He believed, with Dr. White, that the induction of premature labor to be good treatment, but did not think it could be applied indiscriminately to all cases. But 60 pr. ct. show any tendency to hemorrhage before the eighth month, and in others hemorrhage does not occur till labor comes on.

He would induce premature labor a month or six weeks before full period in all cases, when the nature of the hemorrhage clearly indicated the existence of placenta prævia.

Placenta prævia lateralis does not usually give rise to a great amount of hemorrhage. Cases, in which there is a continued leakage from the uterus, can often get along by giving ergot, and when labor comes on and the head presents, the efforts of Nature are usually quite sufficient to complete it. Another point, which should put the physician on his guard, is the liability to post partum hemorrhage, following placenta prævia.

Dr. Hauenstein said he was indebted to Greenhalgh for the idea of covering the rubber bag with cotton, and he believes he also suggested the induction of premature labor, when placenta prævia existed eleven years ago, and when labor comes on and the head presents, let Nature take the natural course.

Dr. Samo said he had been very much interested in Dr. Moody's paper. He inquired if it had been observed by those present that placenta prævia is prone to recur in the same individual.

Dr. Bartlett said his experience had embraced 7 cases, or about 1 in 300 labors. He believed 'that cases of true central placental miflautation were very rare.

He was called not long since to see a Mrs. Foster, on Elk street, who had a midwife in attendance. He found that she had been bleeding profusely; her extremities were cold and her temperature 95½° F. Ergot was at once given and the fingers crowded upwards, through the os uteri, dilating it. He found the child high up in the uterus, and the cord pulseless. The child was brought down at once, and delivered.

A sponge was dipped in a solution of per sulph. of iron, and a cord attached to it, and passed to the fundus of the womb; the uterus immediately contracted. The sponge was afterwards expelled; the temperature rose to 104° F.; the woman recovered.

Mrs. O'Day, on Chicago street, sent for me not long ago in great haste. On my arrival, found her flowing and very weak from loss of blood. The cord was pulseless, and the uterus not inclined to contract. The hand was introduced in the uterus, the child turned and delivered. She made a good recovery. Of the children, all were lost; the mothers were saved.

Dr. Hartwig said he had one case occur in his practice. He would induce premature labor if the patient could be under his constant observation.

For tamponing, he used cotton batting dipped in a solution of tincture of the chloride of iron and water, and dried before using.

As a voluntary communication, Dr. Hartwig reported a successful case of ovariotomy, the lady being pregnant about six weeks at the time she was operated upon. She miscarried at four and a half months, and recovered.

Dr. Bartlett mentioned a young man suffering from typhoid fever, with a temperature of 105° F. He was apparently doing well, when suddenly the temperature fell to 97° F. It remained at this point for several hours, when a large amount of blood was expelled from the bowels. His temperature again went up, and following the use of ergot, tannin, and hydrochloric acid, he recovered. He thought a sudden fall of temperature in this disease might be made a useful diagnostic point in concealed hemorrhage.

Dr. Rochester mentioned the occurrence of urticaria, caused by eating freely of peaches.

Some diphtheria was reported prevalent in the eastern part of the city.

